# Divergent water use strategies of three desert shrubs on the Alxa plateau: insights from stable isotopes and root distribution analysis

**DOI:** 10.3389/fpls.2026.1735586

**Published:** 2026-04-07

**Authors:** Yan-xia Pan, Ai-min Li, Xiong-zhong Ma, Ya-feng Zhang, Xin-ping Wang

**Affiliations:** 1 Shapotou Desert Research and Experiment Station/Key Laboratory of Ecological Safety and Sustainable Development in Arid Lands, Northwest Institute of Eco-Environment and Resources, Chinese Academy of Sciences, Lanzhou, China; 2 College of Urban Construction, Heze University, Heze, China; 3 School of Geographical Science and Planning, Nanning Normal University, Nanning, China

**Keywords:** Alxa plateau, desert shrubs, stable isotopes, water uptake strategies, water use efficiency (wue)

## Abstract

**Introduction:**

Understanding how desert shrubs partition water sources is critical for predicting ecosystem responses to increasing drought and guiding restoration efforts in arid regions. However, the water use strategies of coexisting shrubs in cold deserts remain poorly resolved, particularly under varying soil moisture conditions.

**Methods:**

We quantified the water uptake patterns of three dominant desert shrubs (*Caragana korshinskii, Ammopiptanthus mongolicus*, and *Reaumuria soongarica*) on the Alxa Plateau, China, using stable hydrogen (δD) and oxygen (δ^18^O) isotopes in precipitation, soil water, xylem water, and groundwater, combined with leaf δ^13^C analysis and root distribution measurements. The Bayesian mixing model (MixSIAR) was used to estimate the proportional contributions of six soil layers to plant water uptake under wet, moderate, and dry soil moisture conditions.

**Results:**

The three species exhibited distinct water use strategies. *C. korshinskii* consistently obtained 70-90% of its water from the 40-100 cm soil layer across all moisture conditions, showing the lowest water absorption plasticity (CV=15.5%). *A. mongolicus* shifted flexibly between the 20-60 cm and 60-80 cm layers (CV=16.5%), while *R. soongarica* displayed a bimodal strategy, switching from the 0-20 cm layer under wet conditions to the 60-100 cm layer under drought (CV=16.3%). Leaf δ^13^C values confirmed a depth-water use efficiency (WUE) trade-off, with deeper-rooted species exhibiting higher intrinsic WUE.

**Discussion:**

These findings reveal species-specific water partitioning strategies that reflect differences in root architecture and hydraulic regulation. The results provide a mechanistic basis for targeted ecological restoration: *C. korshinskii* is suited for dune crests with deep stable moisture, *A. mongolicus* for intermediate slopes benefiting from dimorphic roots and nitrogen fixation, and *R. soongarica* for microsites that episodically concentrate surface water. Such species-site matching can enhance restoration success under projected drier climates.

## 1 Introduction

Plants mediate water and energy fluxes in terrestrial ecosystems by coupling soil, vegetation and the atmosphere (Zhang et al., 2020; Dai et al., 2023). Plant–soil water interactions have long been central to ecohydrological research (Hervé-Fernández et al., 2016). Root water uptake sustains transpiration, photosynthesis and metabolism (Zhao and Wang, 2021). Plant water sources vary primarily with root distribution and soil-moisture heterogeneity (Yang et al., 2015; Grossiord et al., 2017). Quantifying species-specific responses to fluctuating water sources is essential for modeling soil–vegetation–atmosphere water fluxes. This understanding is also important to predicting ecosystem structure and function, as well as determining future adaptation strategies under more extreme climatic conditions. In water-limited ecosystems, elucidating species-specific water-use strategies is therefore fundamental not only for advancing plant ecophysiology but also for guiding targeted ecological restoration and combating desertification under a changing climate (Nie et al., 2011; Grossiord et al., 2020).

Climate change is amplifying the frequency and duration of seasonal droughts (Zhang et al., 2020). Although deep soil moisture can sustain some phreatophytes, many desert shrubs depend on episodic surface pulses (Schenk and Jackson, 2002), whereas shallow-rooted species rely on topsoil moisture year-round (Rudov et al., 2023). Consequently, shrubs must adjust their water-use strategies according to varying water availability to ensure survival (Wu et al., 2016; Grossiord et al., 2017). Root architecture determines the depth from which water is extracted. Deep-rooted shrubs access stable soil water, buffering against surface drought (Chimner and Cooper, 2004), thus overcoming the water scarcity in the surface soil layers, which may be subject to frequent drying (Dai et al., 2015). Many shrubs also produce dimorphic root systems that simultaneously exploit shallow and deep water (Dawson and Pate, 1996) and adjust stomatal conductance and leaf water potential (Quero et al., 2011; Altieri et al., 2015) to cope with variations in environmental water sources. These strategies are indicative of the high ecological plasticity and competitive advantages plants possess, which enable them to survive in challenging environments (Antunes et al., 2018; Gao et al., 2018).

Located on the western margin of the East Asian monsoon, the Alxa Plateau receives only 120 mm yr^-1^ precipitation and experiences coefficients of variation in seasonal rainfall exceeding 60% (Wang et al., 2006). The plateau is a major source of dust storms that exacerbate water loss through aeolian erosion (Xiao et al., 2019). Severe desertification therefore makes the plateau a focal region for restoration. In such ecosystems, desert plants—particularly shrubs and dwarf shrubs—play a crucial role in stabilizing soil, preventing erosion, and mitigating desertification. In this context, understanding the dynamics of soil water availability, root distribution, and plant water uptake is essential for enhancing our knowledge of how plants adapt to water-limited environments (Kang et al., 2024).

To identify water sources, researchers have combined root excavation (Xu and Li, 2006), sap-flow sensors (Delzon and Loustau, 2005), electrical resistivity (Mares et al., 2016), GIS tools (Howard and Merrifield, 2010) and even tritium labeling (Zhang et al., 2017). Stable-isotope profiling is now the standard non-destructive tool for partitioning plant water uptake (Ma and Song, 2016; Geris et al., 2017; Hardanto et al., 2017; Rothfuss and Javaux, 2017). Matching xylem and soil δD–δ^18^O profiles quantifies the proportional contribution of each soil layer to plant water (Bello et al., 2019). Stable isotopes in plant leaves also provide valuable insights into the environmental factors affecting plant growth and water use. Isotopic variation in leaf water can reflect changes in temperature (Zhou et al., 2011), relative humidity (Barbour and Farquhar, 2001), vapor pressure deficit (Li et al., 2006), transpiration rate (Sheshshayee et al., 2005), and stomatal conductance (Farquhar et al., 2007). These environmental factors influence the equilibrium fractionation effect, which is further modulated by stomatal conductance and transpiration rates, thereby affecting the isotopic composition of leaf water (Cernusak et al., 2008). Despite the growing body of literature on these isotopic techniques, yet how leaf-water isotopes vary with cold-desert water pulses remains unresolved.

Global syntheses of dual-isotope studies center on Mediterranean and warm deserts, overlooking cold deserts where winter drought coincides with summer pulses (Moreno-Gutiérrez et al., 2012; West et al., 2012). Here we quantify how this bimodal precipitation regime reshapes vertical water partitioning among three co-existing cold-desert shrubs. We (i) quantify proportional water uptake from six soil layers under wet, moderate and dry conditions; (ii) couple Bayesian mixing (MixSIAR) with root architecture and leaf δ^13^C to test the depth–WUE trade-off; and (iii) derive species-specific planting guidelines under SSP5-8.5 drought scenarios.

## 2 Materials and methods

### 2.1 Study area

**Figure 1:**
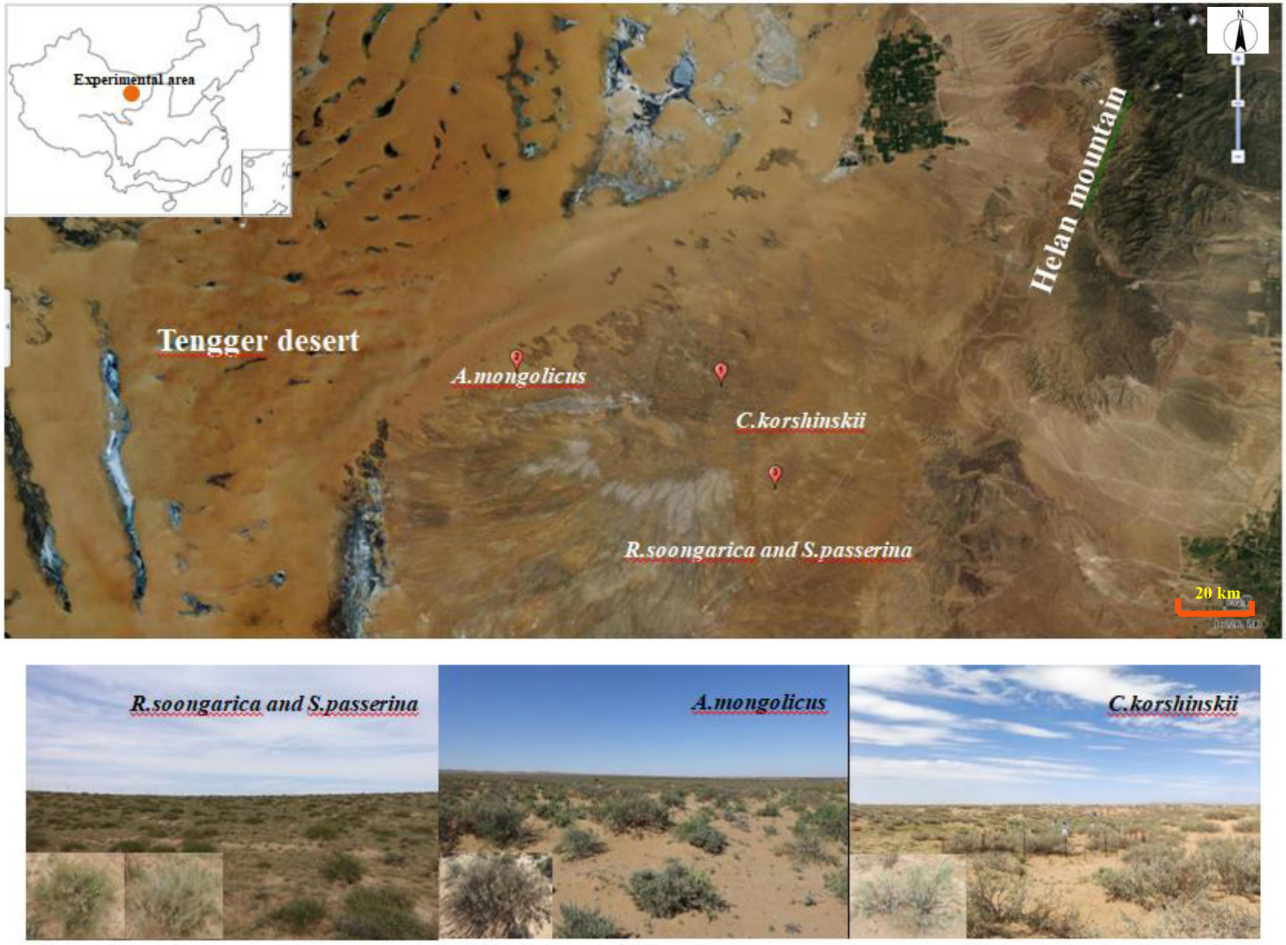
Location and landscape of the experimental sites.

The Alxa Plateau, an ancient eroded high plain, lies in north-western China’s arid interior. It encompasses the Badain Jaran, Tengger and Ulanbuh deserts. Our sites lie on the south-western margin of the Tengger Desert (**Figure 1**), at an elevation ranging from 1300 to 1550 m above sea level. This region is classified as a transitional desert steppe, has a temperate continental arid climate with low precipitation, high sunshine duration, strong winds and scarce surface water.

Mean annual air temperature is 9.4°C and precipitation 119.5 mm (1999–2018) (Ma and Wang, 2020). These conditions render the region highly arid and prone to water scarcity. Furthermore, groundwater is located at depths greater than 40 m below the surface, based on local well measurements. Shrubs therefore rely solely on precipitation and the soil water it recharges. Such limited water availability underscores the importance of understanding plant water use strategies in this environment, where survival is tightly linked to the ability to access and utilize available water sources.

The study area is characterized by the dominance of desert shrubs, which play a critical role in stabilizing soil, preventing erosion, and mitigating desertification. Three typical desert shrub species were selected for this study, all of which are widely distributed in the Alxa steppe desert region (**Figure 1**). These species include *Reaumuria soongarica*, *Ammopiptanthus mongolicus*, and *Caragana korshinskii*. *R. soongarica* (Tamaricaceae family) is a drought-tolerant shrub known for its ability to withstand saline conditions. It is commonly used for soil and water conservation as well as sand fixation, making it a crucial species in desertification control. Its adaptability to extreme drought conditions allows it to thrive in the harsh environment of the Alxa Plateau. It is often characterized by a shallow root system and an opportunistic strategy to capture ephemeral surface moisture. *A. mongolicus* (Leguminosae family) is an evergreen broad-leaved shrub endemic to central Asia, particularly the deserts of northern China. This species is considered rare and endangered due to its limited distribution and ecological significance. As a legume, it also contributes to nitrogen fixation, enhancing soil fertility in desert ecosystems (Xiao et al., 2019). Its dimorphic root system is thought to confer a flexible water-use strategy, allowing access to both shallow and deeper soil water sources. *C. korshinskii* (Leguminosae family) is another drought-resistant shrub that is well-adapted to the arid desert environment. It is a dominant species in fixed and semi-fixed sandy lands and plays an important role in artificial sand fixation projects, which are vital for controlling desertification in arid regions. This species is frequently associated with a deep-rooting habit, enabling it to utilize more stable water reserves from deeper soil layers.

### 2.2 Meteorological and soil moisture measurement

An automatic weather station in each plot recorded air temperature, humidity, wind speed and precipitation. We installed CS655 probes (Campbell Scientific, Logan, UT, USA; ± 0.05% vol.) at 0–20, 20–40, 40–60, 60–80 and 80–100 cm in each plot.

### 2.3 Root distribution measurement

We sampled roots at 10-cm increments to 150 cm with a 10-cm-diameter auger. At each shrub community, three individual plants per species were randomly selected. One core per individual, three individuals per species (n=3). Cores were washed, oven-dried at 65°C to constant mass and weighed to 0.001 g.

### 2.4 Isotope sample collection

We used stratified random sampling in three 1-ha plots (> 20 km apart). Each plot contained three 20 × 20 m subplots separated by 50 m buffers.

In this study, rainwater, soil, plant xylem, and leaf samples were systematically collected to investigate water utilization strategies and isotopic signatures across different components of the ecosystem. The sampling was conducted at multiple time points to capture variation under different hydrological conditions, including periods of varying precipitation and seasonal changes.

Three rain gauges per plot collected precipitation, which was pooled into a single composite sample for isotope analysis.

Soil was sampled in September 2019 (dry), March 2020 (moderate) and July 2021 (wet). Samples were collected with a stainless-steel auger, immediately sealed in glass vials, and stored at -20°C. Soil water was extracted via cryogenic vacuum distillation prior to isotopic analysis. In every plot, soil profiles (n=3 per plot) were excavited adjacent to three randomly selected individuals of the target species, yielding 9 profiles per species in total. For each profile, samples were extracted from six depth intervals: 0–10 cm, 10–20 cm, 20–40 cm, 40–60 cm, 60–80 cm, and 80–100 cm.

Plant Samples were collected from three dominant shrub species. For each species, three replicate individuals were randomly selected, xylem tissue from each species was sampled from the sun-exposed sides of the plants to avoid biases due to shading effects, which can lead to isotopic gradients (Liu et al., 2016). These tissue samples were carefully selected to ensure uniformity in size and physiological state across species, minimizing the potential influence of internal plant variation on isotopic data.

Leaf Samples were also collected to evaluate isotopic differences within the leaf. For each species, three replicate individuals were also randomly selected. As isotopic gradients are often present along the length of a leaf due to differential water uptake and transpiration rates (Farquhar et al., 1989), intact leaves were selected from the middle of the plant canopy to avoid edge effects. The leaves were carefully chosen to be of similar size across species, as leaf morphology can significantly affect the isotopic composition. Groundwater samples were collected from three local wells (>40 m depth) during each sampling campaign to characterize its isotopic composition and exclude it as a direct water source. All field sampling was conducted between 8:00 am and 10:00 am, ensuring consistency in the timing of sample collection to minimize diurnal variability in water isotopic composition.

Upon collection, all samples were immediately transferred to pre-labeled glass bottles, sealed with parafilm to prevent contamination, and stored at -20°C until further analysis. This rapid processing and freezing of samples is critical for preserving the isotopic integrity of the samples before they undergo laboratory analysis (Dawson et al., 2002).

### 2.5 Isotope analyses

Xylem water and soil water samples were extracted using a fully automated vacuum condensation extraction system (LI-2100, LICA United Technology Limited, Beijing, China), with a water extraction efficiency exceeding 98%. This method ensures high precision in the extraction of water from soil and plant tissues, minimizing potential sample loss or contamination during the process.

The hydrogen and oxygen isotope ratios (δ^18^O and δD) of rainwater, soil water, xylem water and groundwater were analyzed using an Isotope Ratio Mass Spectrometer (MAT 253, Thermo Fisher Scientific, Inc., USA). The precision of the isotope measurements was typically better than ±1‰ for δD and ±0.2‰ for δ^18^O, ensuring reliable quantification of isotopic compositions. These isotope ratios were reported in the standard δ-notation, which represents the deviation in parts per thousand (‰) from the Vienna Standard Mean Ocean Water (V-SMOW) reference. The equation used to express these deviations is:


$$\text{δ}\left(‰\right)\;=\;\left({\text{R}}_{\text{sample}}/{\text{R}}_{\text{standard}}-1\right)\;\times \;1000‰$$

where R_sample_ and R_standard_ were the D/H and ^18^O/^16^O molar abundance ratios of the samples and the standard (V-SMOW), respectively. The analyses of each sample were repeated five times and the mean and standard deviation were calculated.

In addition to δD and δ^18^O, the deuterium excess (*d*) was calculated following the method described by Dansgaard (1964):


$$d=\text{ δD}-8\;\times {\text{ δ}}^{18}\text{O}$$

Deuterium excess is a valuable parameter used to assess the degree of evaporation and water source characteristics, providing insights into hydrological processes and climate dynamics.

The carbon isotope ratios (δ^13^C/δ^12^C) in leaf samples were measured using an isotope ratio mass spectrometer (DELTA V Advantage, Thermo Fisher Scientific, Inc., USA). Prior to analysis, the leaf samples were oven-dried at 80°C for 48 hours and finely ground. The relative abundance of δ^13^C and δ^12^C was determined, and the carbon isotope composition (δ^13^C) was calculated using the following formula:


$${\text{δ}}^{13}\text{C}\left(‰\right)\;=\;\left[\left({\text{R}}_{\text{sample}}-{\text{R}}_{\text{standard}}\right)/{\text{R}}_{\text{standard}}\right]\times 1000=\left({\text{R}}_{\text{sample}}/{\text{R}}_{\text{standard}}-1\right)\;\times 1000,$$

where R_sample_ and R_standard_ were the ^13^C/^12^C ratio in the leaf and standard, respectively. The analytical uncertainties for δ^13^C measurements were ±0.1‰.

### 2.6 Analysis method

Descriptive statistics (mean, standard deviation, maximum, minimum) were calculated for the isotopic compositions (δ^18^O and δD) of precipitation, soil water, xylem water, groundwater, and leaf water to characterize their range and distribution under different soil moisture conditions. For each shrub species, three replicate individuals were sampled for xylem and leaf tissues (n=3 per species per sampling campaign), and nine soil profiles (three profiles per plot × three plots) were collected per species (n=9 per depth interval) for soil water isotope analysis. Root biomass was measured from three cores per species (n=3).

Water absorption plasticity (%) is quantified using the coefficient variation (SD/mean, %) of the uptake proportion of each water source (Liu et al., 2021). The mean and SD are the average proportion and standard deviation of plant water uptake to a particular water source during the observation period. Plants with greater water absorption plasticity are more flexible in switching water sources in variable water conditions (Song et al., 2022).

The Bayesian mixing model MixSIAR was used to estimate the proportional contributions of six soil layers (0-10, 10-20, 20-40, 40-60, 60-80, and 80–100 cm) to plant water uptake under wet, moderate, and dry soil moisture conditions. The model was run with three chains, a burn-in of 50000 iterations, and 100000 subsequent iterations, using uninformative prior distributions.

To test for significant differences in water uptake proportions among species within the same soil layer and water regime, as well as differences in leaf δ^13^C values among species under each moisture condition, one-way analysis of variance (ANOVA) was performed, followed by Tukey’s honestly significant difference (HSD) *post hoc* test at α=0.05. Normality and homogeneity of variances were checked using Shapiro-Wilk and Levene’s tests, respectively. Relationships between δ^13^C and maximum rooting depth were examined using linear regression, with significance determined by F-test.

All statistical analyses were conducted in R version 4.2.1. The MixSIAR model was implemented using the R package ‘MixSIAR’ and ANOVA/Tukey HSD tests were performed with base R functions ‘aov’ and ‘TukeyHSD’.

## 3 Results

### 3.1 Vegetation and soil characteristics

*A. mongolicus* communities had the highest canopy cover (31.7%), whereas *R. soongarica* and *C. korshinskii* reached only 9.7% and 19.3%. *C. korshinskii* was tallest (70.3 cm), *R. soongarica* shortest (19.7 cm. *A. mongolicus* canopies were largest, indicating superior light interception (**Table 1**).

**Table 1:** Vegetation and soil characteristics across different community types.

Vegetation community type	*Reaumuria soongarica*	*Ammopiptanthus mongolicus*	*Caragana korshinskii*
Coverage (%)	9.67 (± 0.89)	31.67 (± 2.34)	19.33 (± 1.54)
Plant height (cm)	19.67 (± 1.23)	55.73 (± 3.05)	70.27 (± 3.12)
Canopy (cm)	47.67×46.67 (± 2.38×2.27)	266.55×273.82 (± 11.45×12.34)	181.20×167.47 (± 8.50×7.88)
Soil bulk density (g/cm^3^)	1.45 (± 0.03)	1.57 (± 0.04)	1.62 (± 0.04)
Organic matter (g/kg)	6.65 (± 0.33)	2.94 (± 0.15)	2.21 (± 0.11)
Total Nitrogen (g/kg)	0.57 (± 0.04)	0.30 (± 0.02)	0.22 (± 0.01)
Total Carbon (g/kg)	9.00 (± 0.45)	4.23 (± 0.21)	3.30 (± 0.17)
Total Phosphorus (g/kg)	0.61 (± 0.03)	0.30 (± 0.02)	0.23 (± 0.02)
Available nitrogen (mg/kg)	42.06 (± 2.10)	33.59 (± 1.68)	32.85 (± 1.64)
Available Phosphorus (mg/kg)	12.80 (± 0.64)	5.09 (± 0.25)	7.20 (± 0.36)
Available Kalium (mg/kg)	236.50 (± 11.83)	149.50 (± 7.48)	152.86 (± 7.64)
Sand content (2-0.02mm) (%)	82.84 (± 4.14)	97.78 (± 4.89)	98.15 (± 1.23)
Silt content (0.02-0.002mm) (%)	17.16 (± 0.86)	2.22 (± 0.11)	1.85 (± 0.09)
Clay content (<0.002mm) (%)	4.23 (± 0.21)	0	0

Values in parentheses indicate standard deviation.

Highest bulk density (1.62 g cm^-3^) under *C. korshinskii* reflects tighter packing, potentially reducing infiltration and root penetration. Lowest bulk density (1.45 g cm^-3^) beneath *R. soongarica* enhances aeration and water retention. Soils under *R. soongarica* were most fertile, with highest organic matter (6.65 g kg^-1^), total nitrogen (0.57 g kg^-1^), total carbon (9.00 g kg^-1^), and total phosphorus (0.61 g kg^-1^). This community also has the highest available nitrogen (42.06 mg kg^-1^) and available phosphorus (12.80 mg kg^-1^), which are essential for plant growth. Additionally, *R. soongarica* has the most abundant available potassium at 236.50 mg kg^-1^, a critical nutrient for plant development. *C. korshinskii* soils were least fertile, with lowest organic matter, total nitrogen, carbon, phosphorus, available phosphorus, and a moderate level of available potassium at 152.86 mg kg^-1^. *A. mongolicus* has the lowest available phosphorus at 5.09 mg kg^-1^ and the least available potassium at 149.50 mg kg^-1^, indicating a soil that may not support plant growth as effectively as the *R. soongarica* community.

Sand fraction reached 98% under *C. korshinskii*, favouring rapid drainage but low water storag. *R. soongarica* has the lowest sand content at 82.84% and the highest silt content at 17.16%, which could contribute to better water retention. Clay content is only available for *R. soongarica*, at 4.23%, which can provide nutrients and water retention.

### 3.2 Root distribution characteristics of different plants

**Figure 2:**
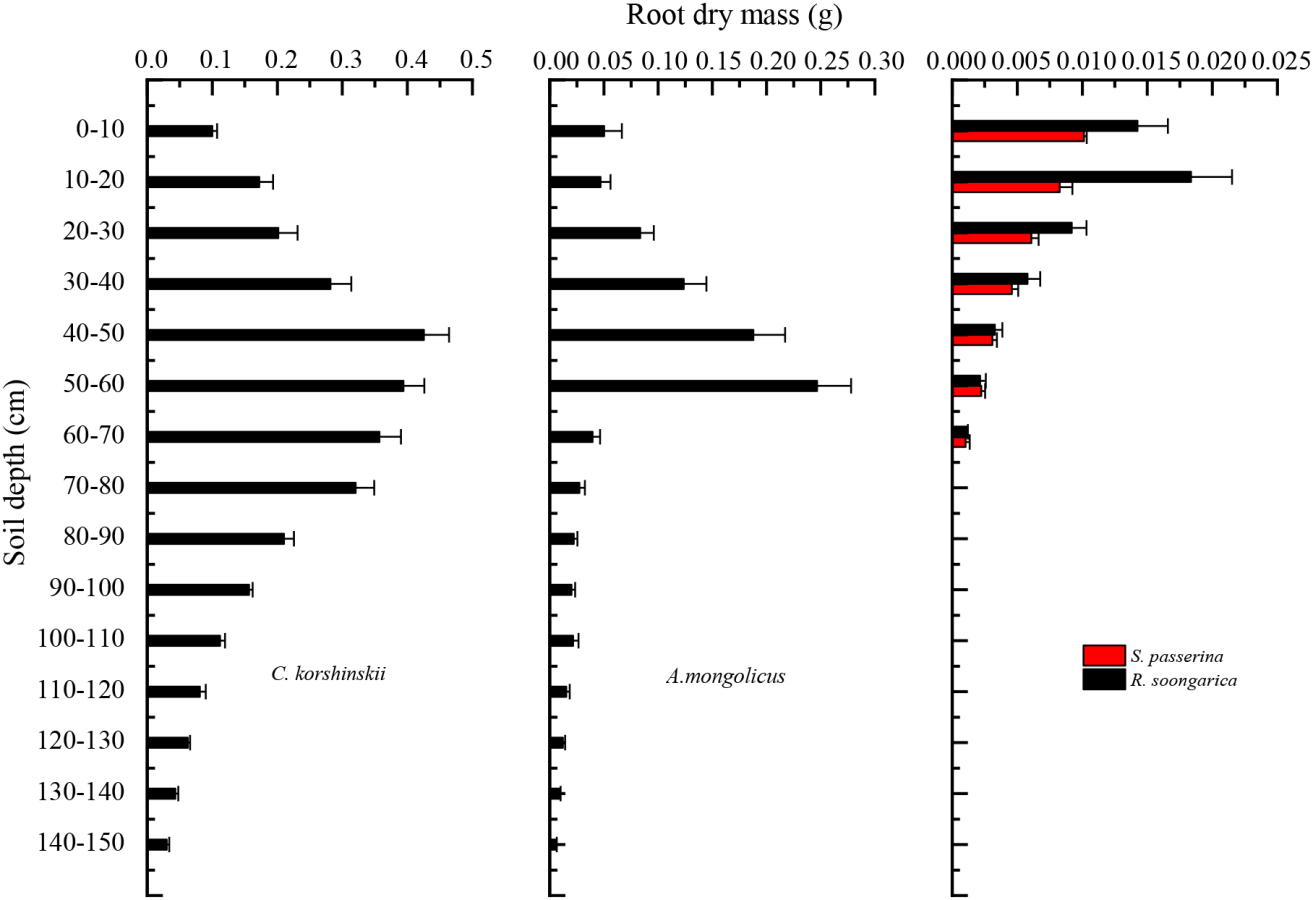
Root dry mass distribution across soil depth intervals for the three shrub species: *C. korshinskii*, *R. soongarica*, and *A. mongolicus*.

Root distribution patterns (**Figure 2**) determine access to water and nutrients. *C. korshinskii* showed uniform root biomass down to 60 cm, with a peak at 40–60 cm. This deep peak allows access to stable soil water, buffering the shrub against surface drought.*A. mongolicus* concentrated 60% of root biomass at 50–60 cm, indicating a dimorphic strategy that can exploit both shallow pulses and deeper reservoirs. The pronounced root biomass in the 50–60 cm layer could be an adaptation to exploit a layer rich in resources, such as moisture or nutrients. The decrease in root mass with depth may indicate a strategy to focus on the most resource-rich layers within the reach of the plant’s root system.

The *R. soongarica* community, in which *Stipa passerina* co-occurs sympatrically, exhibits a bimodal distribution of root dry mass, with pronounced peaks in the 0–10 cm and 10–20 cm soil layers. This pattern indicates a strong presence in the upper soil layers, which is critical for rapid nutrient uptake and anchorage. The decline in root dry mass beyond 20 cm, with a slight resurgence in the 30–40 cm layer, suggests a strategic distribution that balances resource acquisition with energy expenditure. The presence of roots in the 30–40 cm layer could be an adaptation to utilize a secondary resource-rich layer, while the absence in deeper layers may reflect a trade-off between root growth and other metabolic demands.

### 3.3 Soil moisture dynamics

**Figure 3:**
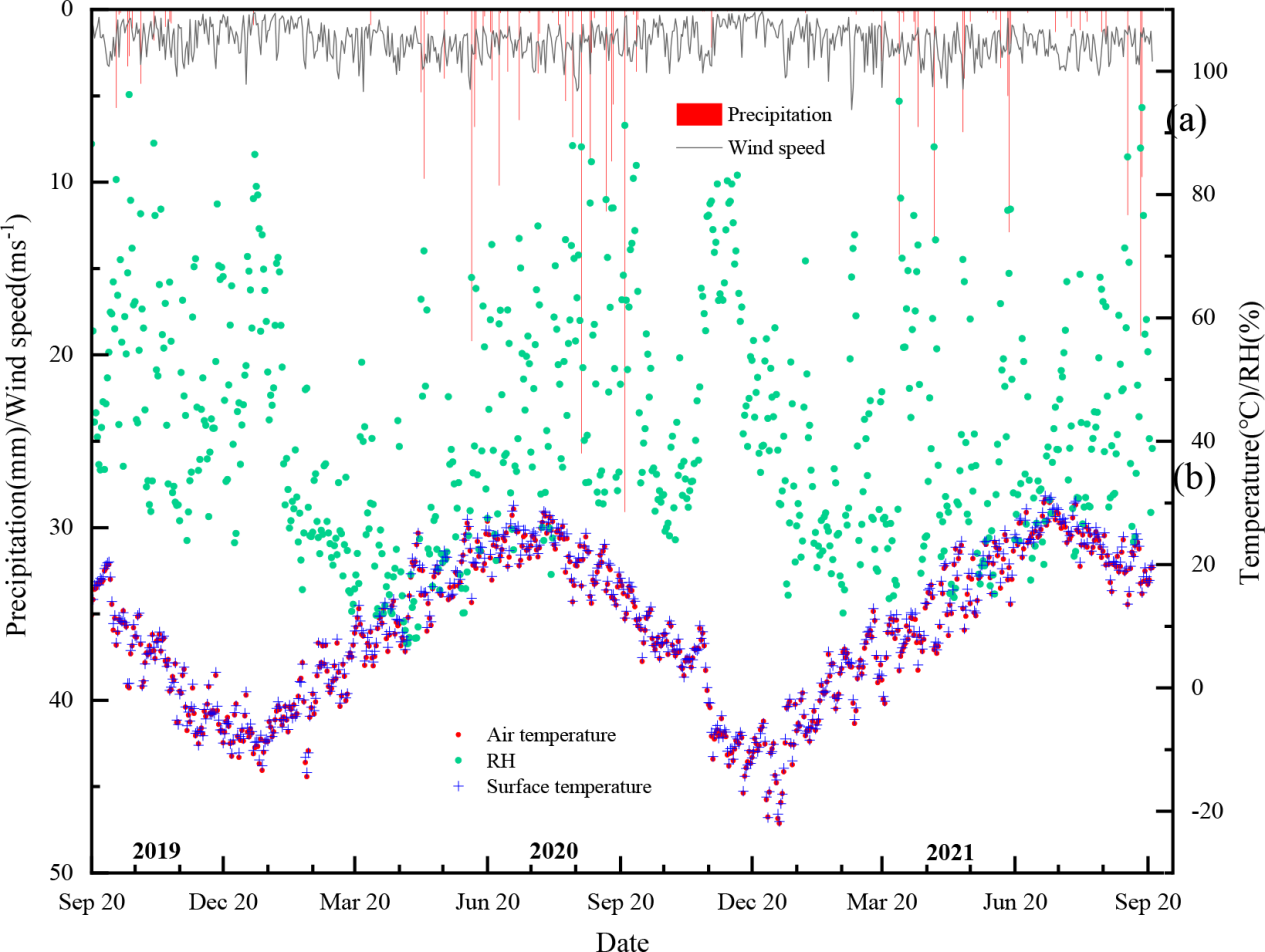
Temporal variations in meteorological factors during the experimental period (*C. korshinskii* community).

**Figure 4:**
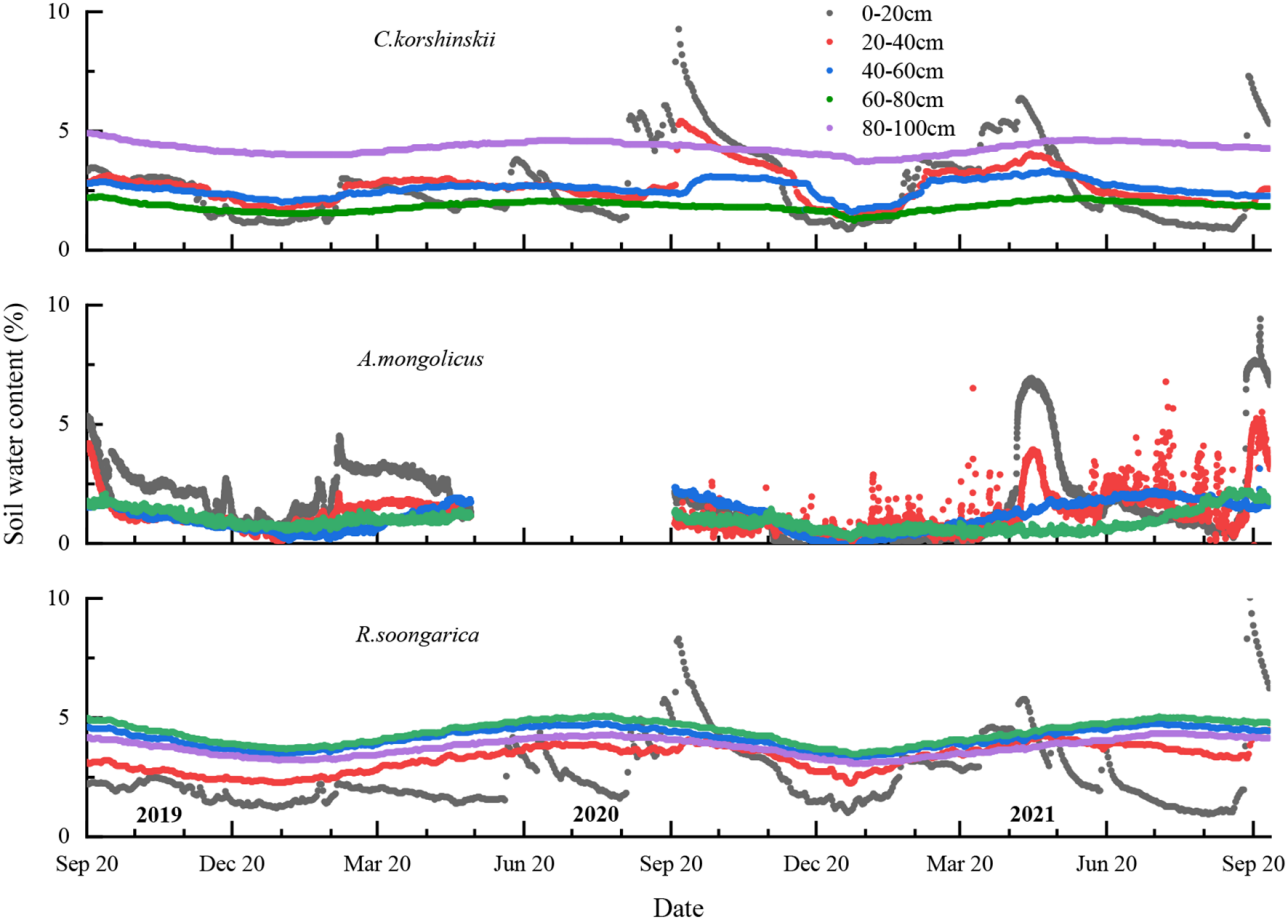
Temporal variations in soil moisture across different plant communities during the experimental period.

**Figure 3** shows daily precipitation, relative humidity and air temperature for the study period. Rainfall peaked during May to September, accounting for 68% of the annual total. These dual pulses replenished the 0–20 cm layer, whereas intervening dry spells depleted soil water below 5% v/v (**Figure 4**).

Soil moisture varied markedly among species and depths (**Figure 4**). For *C. korshinskii*, the top layer (0–20 cm) generally maintains a moderate level of soil water content, which is influenced by direct precipitation and evaporation. Mid-layer (20–60 cm) moisture remained stable (± 0.5% v/v), indicating limited root uptake and low evaporative loss. Deeper layers (60–100 cm) tend to have lower soil water content, which is less affected by short-term weather events and more by long-term climatic conditions. Despite the loss of some data in 2020 due to instrument issues, *A. mongolicus* sample plot exhibits significant fluctuations in the top soil layer (0–20 cm). A sharp drop to 2% in April 2020 was followed by recovery to 6% after 42 mm rainfall, confirming rapid recharge and intensive uptake. Mid layers (20–60 cm) have moderate variations, which may reflect a balance between water input and uptake by the plant’s root system. Deeper layers (60–100 cm) show relatively lower soil water content, which could be indicative of limited water penetration at these depths.

Surface (0–20 cm) moisture under *R. soongarica* peaked at 12% after rainfall, exceeding that of the other two species by 3–4%. Mid layers (20–60 cm) exhibit a decrease in soil water content, which could be influenced by the plant’s root activity and the availability of water. Deeper layers (60–100 cm) maintain a stable soil water content, similar to the other communities, indicating a stable subsurface environment. These soil moisture patterns provide the context for interpreting the divergent water uptake strategies identified in Section 3.5.

### 3.4 Isotopic compositions of different water sources

Precipitation δD ranged from -187 to -7‰ and δ^18^O from -24 to -2‰. The local meteoric water line (LMWL) was δD = 7.91δ^18^O+16.1 (R^2^ = 0.96, *p* < 0.001). Deuterium excess (d=δD-8δ^18^O) averaged 16‰, indicating low relative humidity during precipitation events, typical of cold-desert climates. Soil, xylem and leaf water plotted below the LMWL, evidencing evaporative enrichment. The variation ranges of δD and δ^18^O in soil water are -77.96 to 11.18‰ and -13.05 to 19.58‰. At any given depth, soil-water isotopes did not differ among species (*p*>0.05), confirming homogeneous soil isotopic composition before uptake. The soil water line yielded a slope of 2.75 (R^2^=0.86, *p* < 0.001), consistent with strong evaporative enrichment in surface layers. And that for xylem water are -114.14 to -36.32‰ and -13.64 to 0.04‰, and they are not significantly different for different plants (*p*>0.05), the xylem water line is δD =5.45δ^18^O-34.72 (R^2^ = 0.93, *p* < 0.001).

**Figure 5:**
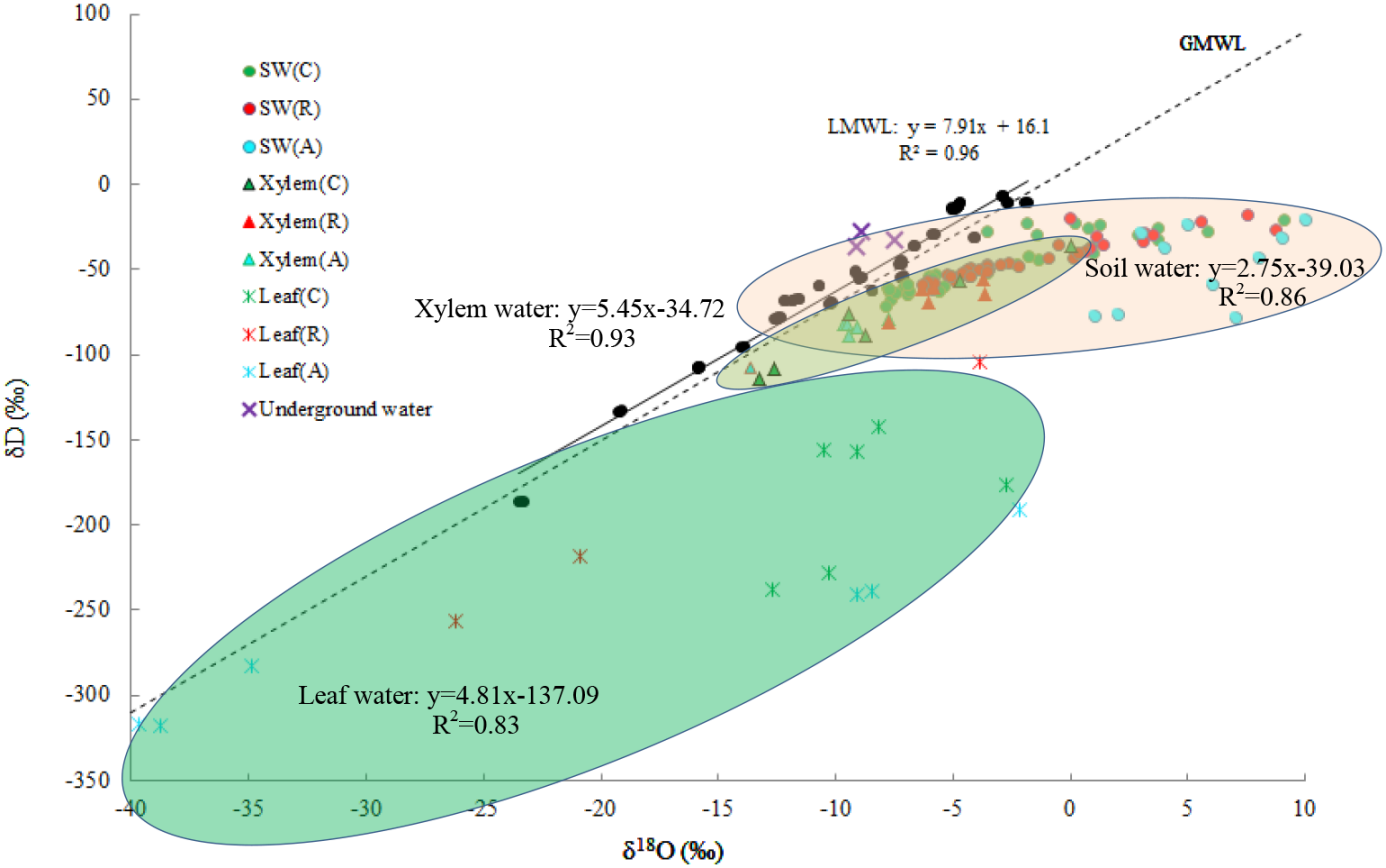
Scatter distribution of δD-δ^18^O in precipitation, soil water, xylem water, and leaf water across the three shrub species. SW(C), SW(R), and SW(A) represent soil water from the *C. korshinskii*, *R. soongarica*, and *A. mongolicus* communities, respectively. Similarly, Xylem(C), Xylem(R), and Xylem(A) denote xylem water from the *C. korshinskii*, *R. soongarica*, and *A. mongolicus* communities, while Leaf(C), Leaf(R), and Leaf(A) indicate leaf water from the corresponding communities.

The mean values of δD and δ^18^O in leaf water are from -318.26 to -38.76‰ and -39.69 to 14.14 ‰, which are significantly poor compared to the xylem water isotope (*p* < 0.05). The leaf water line is δD=4.81δ^18^O-137.09 (R^2^ = 0.83, *p* < 0.001). There was a significant correlation between δ^18^O and δD values for soil water (R^2^ = 0.86, *p* < 0.01), xylem water (R^2^ = 0.93, *p* < 0.01) and leaf water (R^2^ = 0.83, *p* < 0.01). Groundwater plotted above the LMWL, confirming negligible contribution to shrub water uptake.In contrast, the isotope ratios of xylem water match the distribution area of soil water isotope (**Figure 5**), which suggesting that soil water was the primary water source for the shrubs and verifying that no significant isotopic fractionation occurs in these plants during root water uptake as well as water transport.

### 3.5 Variation in the proportion of plant water uptake

**Figure 6:**
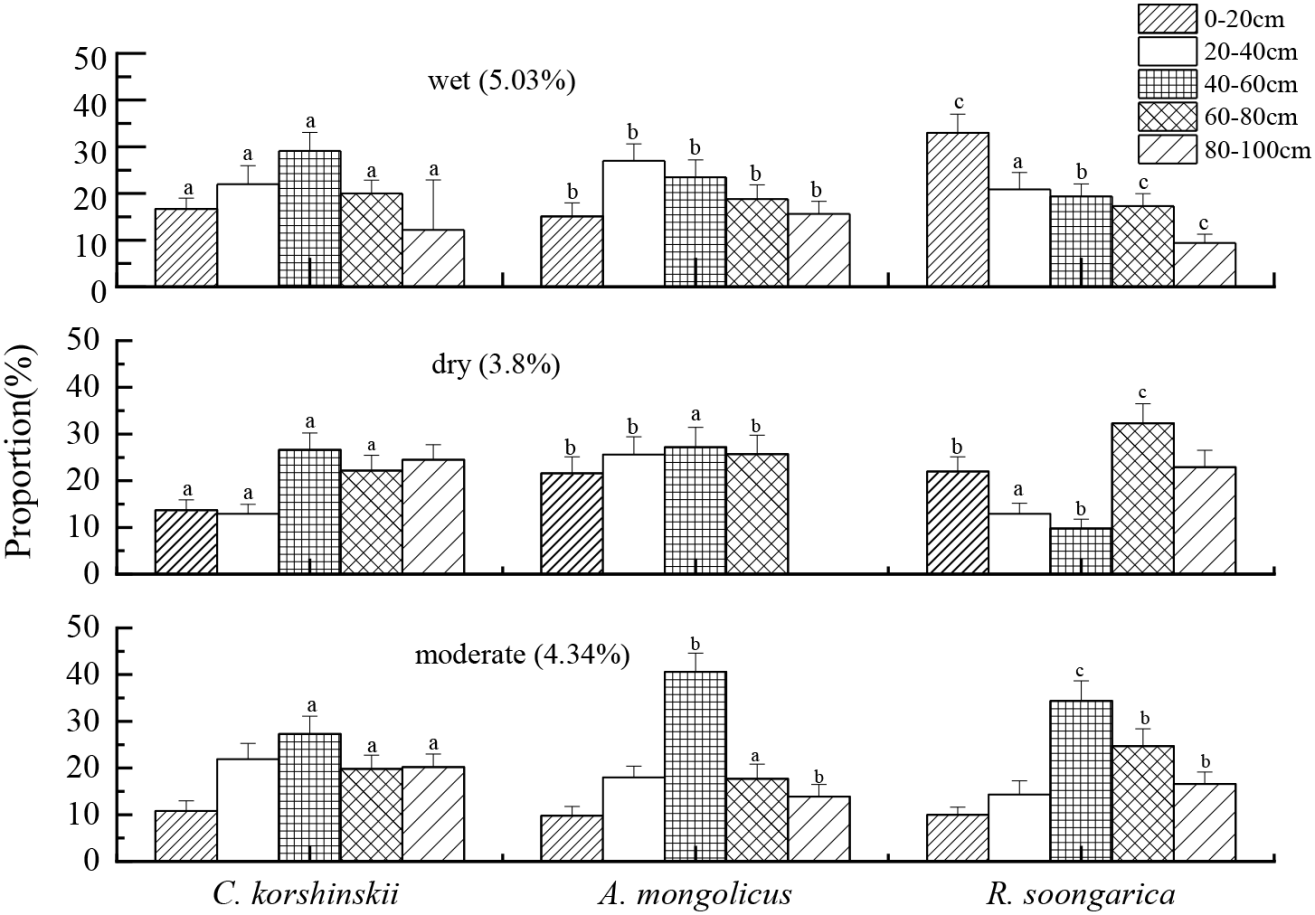
Proportions of water uptake from different soil layers by the three shrub species under varying soil moisture conditions. (Wet condition: measured on September 25, 2020, with 0–100 cm soil water content of 5.03%; dry condition: measured on October 20, 2019, with 0–100 cm soil water content of 3.8%; moderate condition: measured on May 1, 2021, with 0–100 cm soil water content of 4.34%). Lowercase letters above bars denote significant inter-specific differences (Tukey HSD, *p* < 0.05) within the same soil layer and water regime; letters are positioned sequentially in the order *C. korshinskii*, *A. mongolicus* and *R. soongarica* for each depth.

Based on the MixSIAR model, we determined the proportion of water uptake by three shrub species from each soil layer under different soil water conditions, and the results are presented in **Figure 6**. The *C. korshinskii* shrub primarily absorbs water from the soil layers within the 0–80 cm depth under wet conditions. As the soil water content decreases, the primary soil water supply layer gradually deepens. Under moderate water conditions, the 20–100 cm soil layer serves as the primary water-absorbing layer, supplying nearly 90% of the plant’s water source. In dry conditions, 73.3% of the *C. korshinskii* shrub’s water uptake comes from the 40–100 cm soil layer. Moreover, *C. korshinskii* obtained 70–90% of water from 40–100 cm regardless of season, the 40–60 cm layer consistently supplied >50% of uptake. Deep stable uptake minimizes exposure to evaporative loss, explaining the species’ narrow plasticity (CV = 15%).

For *A. mongolicus*, the 20–60 cm soil layer provides more than 50% of the plant’s water uptake sources under wet conditions. Under moderate soil moisture conditions, more than 40% of the plant’s water uptake source is supplied by the 40–60 cm soil layer. Additionally, the proportion of water absorbed from the 60–80 cm soil layer under dry conditions is higher than that under wet and moderate conditions.

In wet conditions, the 0–20 cm soil layer is the primary water uptake layer for *R. soongarica*, supplying 33% of the plant’s water source. Under moderate water conditions, the proportion of water absorbed by the plant from the 40–60 cm layer is more than 34%. In dry conditions, the 60–100 cm soil layer supplies more than 50% of the water uptake source for *R. soongarica*, which is significantly higher than that under wet and moderate conditions (*p* < 0.05).

**Figure 7:**
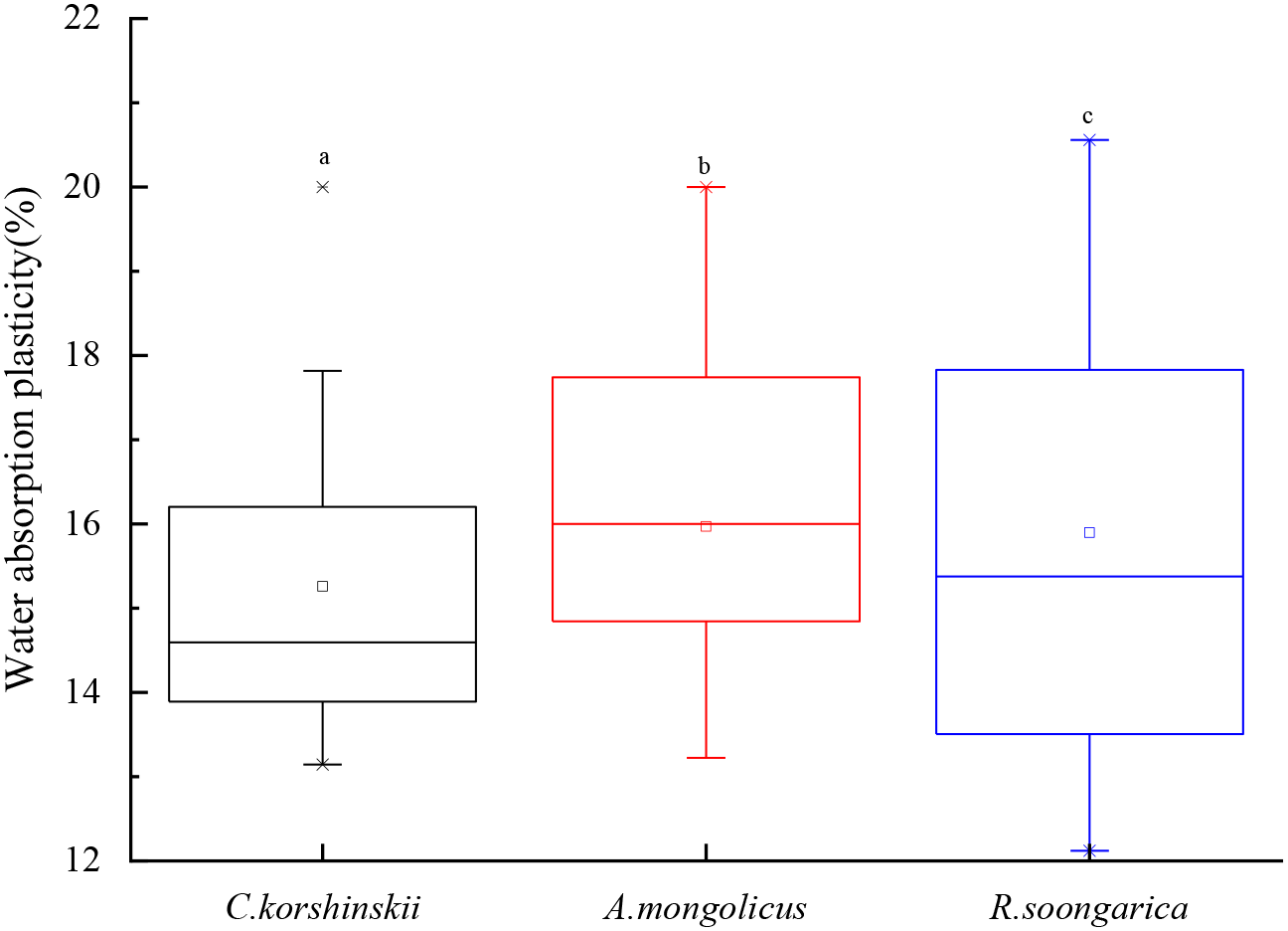
Water absorption plasticity of the three shrub species during the observation period. The boxes show the interquartile range (IQR; 25th to 75th percentile), the central line indicates the median, and the whiskers extend to the 5th and 95th percentiles. Different lowercase letters above boxes indicate significant inter-specific differences (Tukey HSD, *p* < 0.05).

The analysis of water absorption plasticity, as depicted in **Figure 7**, reveals significant variations among the three dominant shrub species. *C. korshinskii* showed the lowest plasticity (CV = 15.5%), consistent with deep stable uptake. The constrained variability and few outliers suggest a stable and uniform adaptation mechanism to water uptake. In contrast, *A. mongolicus* shows greater variability with a median plasticity of around 16.5% and a wider IQR from 14.75% to 18.75%, indicating greater adaptability via dimorphic roots. Notably, *R. soongarica* demonstrates the highest plasticity variability, with a median of 16.25% and an extensive IQR ranging from 14% to 19%, along with significant outliers at both the high (21%) and low (12.5%) ends. This broad range reflects high plasticity conferred by shallow cluster roots plus deep secondary roots. The comparative analysis underscores that while *C. korshinskii* shows minimal variability, *A. mongolicus* and *R. soongarica* possess more flexible water absorption strategies, potentially enhancing their resilience in arid environments.

### 3.6 Variation in leaf δ^13^C values

**Figure 8:**
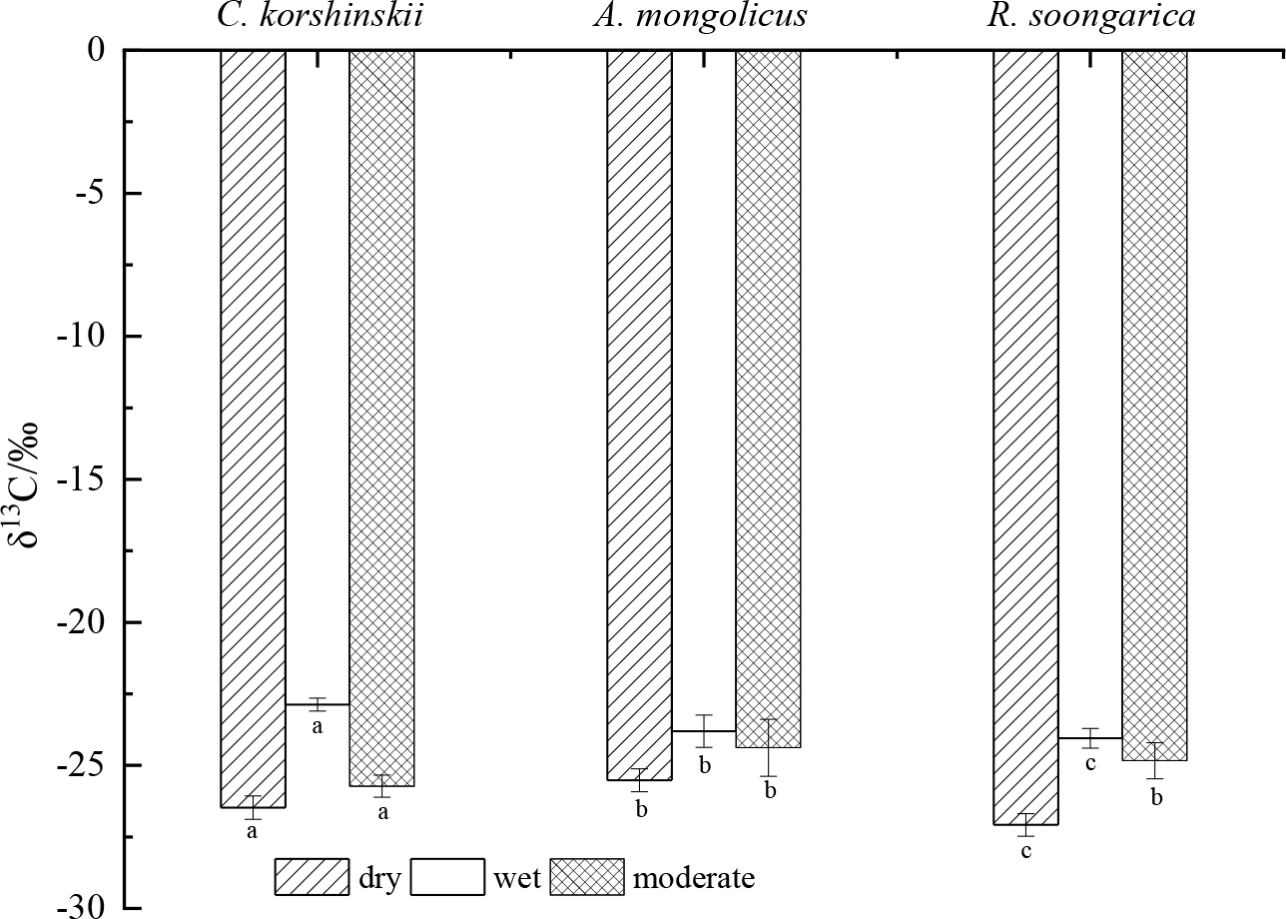
Variation in δ^13^C values of the three shrub species under different soil moisture conditions. (Wet condition: measured in Sep. 25th, 2020, with 0–100 cm soil water content of 5.03%; dry condition: measured on October 20, 2019, with 0–100 cm soil water content of 3.8%; moderate condition: measured on May 1, 2021, with 0–100 cm soil water content of 4.34%). Different lowercase letters above bars indicate significant inter-specific differences within each water regime (Tukey HSD, *p* < 0.05).

Leaf δ^13^C differed among species (*p* < 0.05), with deeper-rooted species displaying higher intrinsic WUE (**Figure 8**). During drought, *A. mongolicus* maintained the highest δ^13^C (-25.8‰), consistent with accessing deeper, more reliable water. In wet periods, *C. korshinskii* retained high δ^13^C (-24.9‰), reflecting conservative stomatal control. Under moderate condition, although *A. mongolicus* still has higher δ^13^C values than the other two species, the differences are not statistically significant (*p*>0.05). These δ^13^C variations reflect intrinsic water-use efficiency (WUE) differences among species, with higher δ^13^C values indicating greater WUE, particularly under drought stress (Farquhar et al., 1989). Across all conditions, δ^13^C increased linearly with maximum rooting depth (R^2^=0.72, *p* < 0.01), supporting a universal depth-WUE trade-off.

## 4 Discussion

The findings of this study provide a comprehensive understanding of the water use strategies employed by three dominant desert shrub species (*C. korshinskii, A. mongolicus, and R. soongarica*) in the arid Alxa Plateau, China. By integrating stable isotope analysis, root distribution data, and soil moisture dynamics, we have elucidated the complex interactions between plants and their water sources in a highly water-limited environment.

### 4.1 Soil water dynamics and plant water uptake patterns

Deep-rooted *C. korshinskii* buffered seasonal drought by extracting 70-90% water from 40–100 cm (**Figure 6**), thereby minimising xylem tension and cavitation risk (Huang et al., 2024). The ability of *C. korshinskii* to shift its water uptake to deeper layers as surface soil moisture decreases is a key adaptation to water scarcity, consistent with findings from other arid regions (Grossiord et al., 2020).

Dimorphic roots allow *A. mongolicus* to switch uptake depth within 48 h after rainfall, maximising resource use efficiency. This adaptability is likely facilitated by its dimorphic root system, which allows it to exploit both shallow and deep soil moisture (Dawson and Pate, 1996; Liu et al., 2021). Recent studies have shown that dimorphic root systems are particularly advantageous in arid environments, as they enable plants to rapidly respond to sporadic rainfall events while maintaining access to deeper water reserves during dry spells (Xu et al., 2016).

Bimodal roots enable *R. soongarica* to exploit surface pulses (<24 h) while maintaining access to deeper reserves. Such bimodal root systems are common in desert shrubs and have been shown to enhance water use efficiency by allowing plants to capitalize on both short-term rainfall events and long-term soil moisture reserves (Antunes et al., 2018). Matching δD-δ^18^O signatures between xylem and sub-soil confirm this depth as the primary water source (**Figure 5**), validating a safety-oriented strategy.

The xylem water isotopic signatures closely match those of soil water, confirming that soil water is the primary water source for these shrubs (**Figure 5**). This finding is consistent with recent studies that have used stable isotopes to trace water sources in arid ecosystems (Rothfuss and Javaux, 2017). The lack of significant isotopic fractionation during root water uptake and transport further supports the reliability of stable isotopes as a tool for studying plant-water interactions in arid environments (Barbeta et al., 2019; Zhao and Wang, 2021).

The deuterium excess (*d*) values, calculated as *d*=δD-8×δ^18^O, provide additional insights into the evaporation processes affecting soil water. The lower *d* values in soil water compared to precipitation indicate significant evaporation enrichment, particularly in the surface soil layers (**Figure 5**). This is consistent with the findings of recent studies that have demonstrated the role of evaporation in altering the isotopic composition of soil water in arid regions (Sprenger et al., 2019). The higher *d* values in deeper soil layers suggest less evaporation influence, which aligns with the observed water uptake patterns of the shrubs, particularly their reliance on deeper soil layers during dry periods.

**Figure 9:**
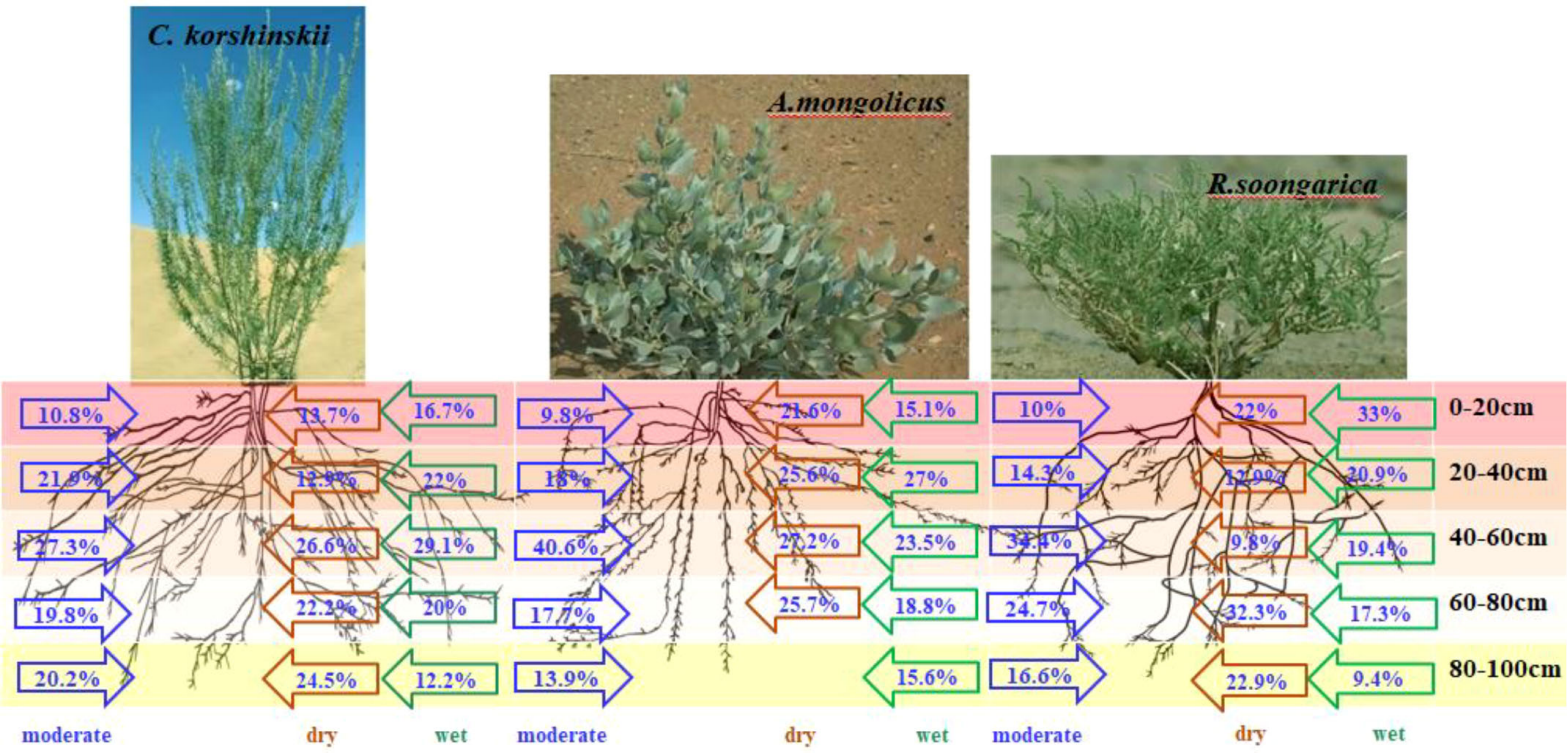
Water uptake patterns of three desert shrubs (*C. korshinsk*ii, *A. mongolicus*, and *R. soongarica*) under wet, moderate, and dry soil moisture conditions on the Alxa Plateau.

The close match between xylem and soil water isotopes, coupled with the distinct root architectures (**Figure 2**), provide a mechanistic basis for the observed source partitioning: deep roots (*C. korshinskii*) minimize exposure to evaporative enrichment, dimorphic roots (*A. mongolicus*) allow integration of multiple sources, and shallow roots (*R. soongarica*) lead to greater uptake of isotopically enriched surface water during wet periods. These contrasting root architectural patterns are illustrated in **Figure 9**, which presents the root distribution profiles and corresponding photographs of the three shrub species.

### 4.2 Water absorption plasticity and ecological implications

*A. mongolicus* and *R. soongarica* show higher variability in water absorption plasticity, suggesting greater flexibility in their water uptake strategies. This flexibility may enhance their resilience to fluctuating water availability, a critical trait in arid environments where water resources are highly variable (Grossiord et al., 2020).

Consistently, we confirm a global depth-WUE trade-off: deeper roots reduce evaporative loss and sustain higher leaf δ^13^C. In Mediterranean shrublands, Moreno-Gutiérrez et al. (2012) showed that co-occurring woody species partition soil water vertically, with deeper-rooted plants exhibiting higher δ^13^C values. Comparable patterns were reported in Iranian gypsum deserts, where shrubs with deeper water access displayed greater WUE (Rudov et al., 2023). Similarly, early work in Australian arid ecosystems (Allison et al., 1983) and recent global syntheses (Muñoz-Gálvez et al., 2025) corroborate that deeper-rooted taxa consistently maintain higher δ^13^C, highlighting a universal depth–WUE trade-off.

High plasticity in *R. soongarica* arises from shallow cluster roots plus facultative deep roots. (**Figure 2**). This adaptability may explain its dominance in certain areas of the Alxa Plateau, where soil moisture conditions can vary significantly over short time scales. The ability to switch between shallow and deep water sources is a key survival strategy for desert shrubs, particularly in regions prone to seasonal droughts (Zhang et al., 2020).

Across global drylands, differential water-use strategies have repeatedly emerged as a fundamental axis of plant coexistence (Schenk and Jackson, 2002). Our findings for the Alxa Plateau add a cold-desert example to this growing body of evidence.

Vertical partitioning of soil water has been documented in Mediterranean shrublands (Moreno-Gutiérrez et al., 2012; Illuminati et al., 2022), Iranian gypsum deserts (De la Puente et al., 2021; Rudov et al., 2023), South-African semi-deserts (February et al., 2013), and Chinese temperate deserts (Xu and Li, 2006; Xu et al., 2007; Wu et al., 2014; Tiemuerbieke et al., 2018). Consistently, deeper-rooted shrubs in these systems display higher intrinsic water-use efficiency (δ^13^C) relative to shallow-rooted congeners (West et al., 2012; Pivovaroff et al., 2016), reflecting a universal depth-WUE trade-off (Muñoz-Gálvez et al., 2025). We observe the same pattern on the Alxa Plateau: *C. korshinskii*, with its deepest root biomass at 40–60 cm and access to 60–100 cm soil water, maintained significantly higher δ^13^C under moderate-to-dry conditions than *R. soongarica*, which concentrated >70% of its roots in the upper 20 cm and shifted opportunistically between surface and deeper sources.

This concordance extends to the plasticity continuum. Kulmatiski et al. (2020) reported that shrubs with dimorphic root systems (e.g., *Artemisia tridentata*) exhibited greater water-source plasticity than strictly deep- or shallow-rooted species. *A. mongolicus* had widest plasticity (IQR 4%), reflecting dimorphic root advantage.Previous isotope studies on *C. korshinskii* in the Loess Hills (Zhang et al., 2020) and *R. soongorica* in the Gurbantonggut Desert (Dai et al., 2015) likewise documented deep-water dependence and surface-water flexibility, respectively. The present study integrates these species within a single environmental matrix, demonstrating that the same depth-WUE trade-off operates along a continuous soil-moisture gradient.

Moreover, gypsum-affinity studies (Palacio et al., 2017; Sánchez-Martín et al., 2021) indicate that edaphic specialization can further refine water-use niches without constraining responses to general drought stress. Our Alxa site, with sandy soils and negligible gypsum, confirms that depth-based partitioning is robust across substrate types.

Thus, vertical water partitioning is a general mechanism allowing coexistence in cold deserts. Deep roots minimise hydraulic failure, shallow roots maximise carbon gain—an efficiency-safety trade-off. Our data reinforce this generality and provide quantitative benchmarks for predicting community responses to intensified drought under future climates (Chen et al., 2024; Fan et al., 2025).

### 4.3 Implications for ecological restoration and climate change adaptation

The findings of this study have important implications for ecological restoration efforts in arid and semi-arid regions. The ability of *C. korshinskii* to access deep soil water reserves makes it a suitable candidate for reforestation projects in areas with limited surface water availability. Similarly, the flexible water uptake strategies of *A. mongolicus* and *R. soongarica* suggest that these species may be well-suited for restoration projects in regions with highly variable precipitation patterns (Nie et al., 2011).

The variations in δ^13^C values reflect differences in water use efficiency (WUE) among the species, with higher δ^13^C values typically indicating higher WUE (Farquhar et al., 1989). The results suggest that *A. mongolicus* and *C. korshinskii* may have more efficient water use strategies under varying soil moisture conditions, while *R. soongarica* exhibits lower WUE, likely due to its reliance on shallow soil water.

The significantly higher δ^13^C values in *A. mongolicus* during dry and moderate condition suggest that this species maintains higher water use efficiency (WUE) under drought conditions, likely due to its deep-rooted system and ability to access deeper soil water reserves (Dawson and Pate, 1996). This is consistent with previous studies that have linked higher δ^13^C values to increased WUE in plants under water-limited conditions (Farquhar et al., 1989; Wang et al., 2005).In contrast, the lower δ^13^C values in *R. soongarica* indicate lower WUE, which may be attributed to its reliance on shallow soil water, particularly during wet conditions. This species likely experiences higher transpiration rates and stomatal conductance, leading to less efficient water use (Farquhar et al., 1989). The higher δ^13^C values in *C. korshinskii* during moderate condition suggest a shift in its water use strategy, possibly due to changes in soil moisture availability. This species may employ a more conservative water use strategy, closing stomata during periods of water stress to reduce water loss.

The more negative δ^13^C during the dry season (**Figure 8**) likely reflects reduced photosynthetic capacity under combined cold and drought stress, consistent with metabolic down regulation observed in winter desert shrubs (Xu and Li, 2006).

Leaf water stable isotopes provide a time-integrated proxy for transpiration fluxes and evaporative demand (Cernusak et al., 2008). The pronounced enrichment observed relative to xylem water (**Figure 5**) reflects the combined effects of high leaf-to-air vapour pressure deficit and sustained transpiration under low soil matric potentials typical of the Alxa Plateau (Barbour and Farquhar, 2001). The absence of species-level differences in the magnitude of ΔδD_leaf-xylem suggests all three shrubs experience similar atmospheric demand once water reaches the leaves, regardless of rooting depth.

Under SSP5-8.5 (+2°C, -15% precipitation, +25% dry-spell length). Restoration must match species to hydrological microsites instead of one-size-fits-all planting. Plant *C. korshinskii* on dune crests where deep moisture persists >40 m above groundwater (Xu et al., 2007). Use *A. mongolicus* on intermediate slopes where episodic recharge allows dual-depth uptake and N input (Xiao et al., 2019). Deploy *R. soongarica* in micro-catchments that concentrate runoff on saline or hard-pan soils (Fan et al., 2025).

As climate change continues to alter precipitation patterns and increase the frequency of extreme drought events, understanding the water use strategies of desert shrubs becomes increasingly important. The insights gained from this study can inform the selection of plant species for large-scale ecological restoration projects, ensuring that the chosen species are well-adapted to the specific water dynamics of the target region. Furthermore, the integration of stable isotope techniques with traditional ecological methods can enhance our ability to predict and manage plant-water-soil interactions in arid ecosystems, contributing to more effective ecosystem management and climate change adaptation strategies.

In summary, (i) deep roots secure hydraulic safety and high intrinsic WUE, (ii) dimorphic or bimodal roots maximise temporal flexibility, and (iii) these strategies can be translated into microsite-specific planting: deep crests for C. korshinskii, recharge slopes for A. mongolicus, and runoff micro-catchments for R. soongarica under SSP5-8.5 drought.

## 5 Conclusion

By coupling triple-isotope profiling with root architecture we unveil a general depth–WUE trade-off in cold-desert shrubs: deep roots secure hydraulic safety and high δ^13^C, shallow roots secure carbon gain but low δ^13^C. Quantitative uptake maps reveal *C. korshinskii* as deep-water specialist, *A. mongolicus* as dimorphic switcher and *R. soongarica* as bimodal opportunist. Under SSP5-8.8 drought, planting should match these hydraulic niches-deep crests for *C. korshinskii*, recharge slopes for *A. mongolicus*, runoff micro-catchments for *R. soongarica*—boosting restoration success and desertification control.

## Data Availability

The raw data supporting the conclusions of this article will be made available by the authors, without undue reservation.
